# Acceptability of Telegenetics for Families with Genetic Eye Diseases

**DOI:** 10.3390/genes12020276

**Published:** 2021-02-15

**Authors:** Suzannah Bell, Urvi Karamchandani, Kirsten Malcolmson, Mariya Moosajee

**Affiliations:** 1 Moorfields Eye Hospital NHS Foundation Trust, London EC1V 2PD, UK; Suzannah.bell@nhs.net (S.B.); u.karamchandani1@nhs.net (U.K.); Kirsten.malcolmson@nhs.net (K.M.); 2 Institute of Ophthalmology, University College London, London EC1V 9EL, UK; 3 Great Ormond Street Hospital for Children, London WC1N 3JH, UK; 4 The Francis Crick Institute, London NW1 1AT, UK

**Keywords:** telemedicine, telegenetics, remote consultations, ophthalmology, service delivery, genetic eye disease, rare disease

## Abstract

Healthcare providers around the world have implemented remote routine consultations to minimise disruption during the COVID-19 pandemic. Virtual clinics are particularly suitable for patients with genetic eye diseases as they rely on detailed histories with genetic counselling. During April–June 2019, the opinion of carers of children with inherited eye disorders attending the ocular genetics service at Moorfields Eye Hospital NHS Foundation Trust (MEH) were canvassed. Sixty-five percent of families (*n* = 35/54) preferred to have investigations carried out locally rather than travel to MEH, with 64% opting for a virtual consultation to interpret the results. The most popular mode of remote contact was via telephone (14/31), with video call being least preferred (8/31). Hence, 54 families who had received a telephone consultation mid-pandemic (November 2020–January 2021) were contacted to re-evaluate the acceptability of telegenetics using the Clinical Genetics Satisfaction Indicator and Telemedicine Satisfaction Questionnaire. Overall, 50 carers participated (response rate 93%); 58% of participants found teleconsultations acceptable and 54% agreed they increased their access to care, but 67.5% preferred to be seen in person. Patient satisfaction was high with 90% strongly agreeing/agreeing they shared and received all necessary information. Ocular genetics is well-suited for remote service delivery, ideally alternated with face-to-face consultations.

## 1. Introduction

In line with the UK response to the COVID-19 pandemic, the government advised that healthcare providers should roll out remote outpatient consultations using video, telephone, email and text message services [1]. The potential role of telemedicine has previously been described in the response to disasters and public health emergencies including COVID-19 [2,3]. Moorfields Eye Hospital NHS Foundation Trust (MEH) is leading a taskforce that is supporting acute providers to rapidly implement virtual consultations where possible, in order to continue the delivery of ophthalmology care during the crisis [1].

Ophthalmology accounts for the largest proportion of outpatient visits per year in the National Health Service (NHS). At MEH, 600,000 outpatients attendances were recorded in 2018/19 and 1% of these were genetic eye disease consultations [4]. As a tertiary referral specialist hospital, referrals come from across the country and many patients maintain dual care with local hospital eye services. This can result in several hospital appointments per year, time off from work or school, travel expenditure and duplication of routine serial monitoring investigations. The increasing prevalence of chronic conditions is necessitating the redesign of patient pathways to improve capacity [5]. Consultations, often described as telemedicine, have been found to be most effective in specialties that primarily rely on verbal interaction for assessment. This makes them highly suitable for genetic eye disease consultations where detailed history-taking and genetic counselling are key features [6]. In ophthalmology, virtual consultations are established in various specialties. At MEH, existing electronic resources including Patient Administration System, Medisoft and intranet based worklists, created using Microsoft SQL Server Reports Software, are used to facilitate virtual consultations in medical retina care [7]. Similarly, in glaucoma care, “virtual clinic modules” created on the existing electronic patient record system (OpenEyes system) are used. This has reduced patient journey times and is highly rated by service users (<3% of respondents (*n* = 620) rated the service as “poor”) [8].

Following the UK 100,000 Genomes Project (an initiative to sequence the genomes of 85,000 NHS patients with rare diseases and cancer to advance diagnosis and develop personalised treatments, and to introduce genomic medicine into our healthcare system), access to genetic testing has changed significantly [9]. There is a national directory of approved genetic tests, three appointed laboratory providers of specialised ophthalmic genomic testing and emerging centralised funding for most rare disease patients. This is yielding higher diagnostic rates, and hence, capacity building is required to facilitate the greater demands on specialist services. The Royal College of Ophthalmologists have issued genomics services guidance that outlines NHS England’s long-term plan to offer whole-genome sequencing (WGS) to 500,000 individuals by 2023 and are focused on integrating genomics into mainstream ophthalmic practice [10]. With this in mind, it has also been recognised that much training and support will be required to general ophthalmologists, and virtual consultations with specialists in this field will be required until the level of competency is reached.

Recent technological advances have resulted in the development and wide-scale implementation of various modalities enabling ophthalmologists to manage patients remotely. These are used mainly to screen retinal conditions such as retinopathy of prematurity, diabetic retinopathy and age-related macular degeneration, diagnose anterior segment conditions and manage patients with glaucoma [11]. The COVID-19 pandemic poses specific challenges to ophthalmology service delivery with social distancing, frequent changes in restrictions, isolation and quarantine periods and patient anxieties around contracting coronavirus affecting both acute and routine patient care. The effects of delayed acute presentations have already been widely reported with the impact on chronic conditions beginning to emerge [12,13,14,15].

Prior to the COVID-19 pandemic, active canvassing of service user opinions and planning for remote genetic eye disease clinics at MEH was underway. Post-implementation, service user satisfaction and acceptability were evaluated to ensure changes were successful in the long-term. The General Medical Council (GMC) standard criteria for appropriateness of remote consultations (Box 1) suggest that patients with genetic eye diseases are suitable for telegenetics [16]. Mainstay of management is genetic testing to determine the cause with pre- and post-test genetic counselling, and subsequent long term follow up to monitor disease progression with widely available imaging that can be done at their local hospital such as colour fundus photography, visual fields testing, spectral domain optical coherence tomography (SD-OCT) and fundus autofluorescence. Most patients with or at risk of developing ocular co-morbidities such as glaucoma or corneal keratopathy are under regular follow-up with the relevant clinical specialty. Paediatric patients can be under several specialists including paediatricians and general paediatric ophthalmologists to ensure their vision is developing and there are no added amblyogenic factors. This may all involve several visits to the hospital per year, which can cause difficulty with taking time off school or work for carers. There are very few specialist genetic eye disease services across the country, which means that remote populations may not have easy access to them [17]. Furthermore, virtual consultations may also reduce the number of sight-impaired patients travelling long distances. Any services implemented must be acceptable to their users; we present our model of setting up remote clinics for our ocular genetics services and our patient satisfaction and acceptability findings.

Box 1 **GMC ethical guidance for remote consultations. Is a remote consultation appropriate? [17].**

**Remote consultations may be appropriate when.**

- The patient’s clinical need or treatment request is straightforward
- You can give patients all the information they want and need about treatment options by phone, internet, or video link
- You have a safe system in place to prescribe
- You have access to the patient’s medical records
- You don’t need to examine the patient
- The patient has capacity to decide about treatment

**Face to face consultations may be preferable when**

- The patient has complex clinical needs or is requesting higher risk treatments
- You are not the patient’s usual doctor or GP and they have not given you consent to share their information particularly if the treatment needs follow up or monitoring
- You do not have access to the patient’s medical records
- You need to examine the patient
- It’s hard for you to ensure, by remote means, that the patients have all the information they want and need about treatment options
- You are unsure of the patient’s capacity to decide about treatment

## 2. Materials and Methods

Prospectively, between April and June 2019, 56 sequential adult carers of paediatric patients attending the ocular genetics service at MEH were given a short questionnaire, approved by the local Patient Experience Committee (Table 1). Participants were asked to indicate their preference of (1) having their tests carried out at a local hospital rather than at MEH and (2) a review of their results virtually rather than physically attending an appointment. If respondents answered positively to a virtual discussion, they were then asked to indicate whether they would prefer a discussion by telephone call, video call or by letter. Participants were asked what proportion of their appointments they would like to be virtual, including options for every or alternate appointments or until a treatment or trial becomes available (where a more complex discussion might be required). Carers indicated a preference for telephone (*n* = 14/31) rather than video (*n* = 8/31) consultations or written communication (*n* = 10/31) at this stage. So when remote consultations were mandated during the pandemic, this mode of contact was utilised more than video consultation for the paediatric cohort.

Subsequently, between November 2020 and January 2021, sequential adult carers of paediatric patients who had recently received a telephone consultation from the ocular genetics service were contacted, via telephone, and asked a short questionnaire about their experience. Patient satisfaction with both genetic counselling and telemedicine was measured using two previously validated questionnaires: Clinical Genetics Satisfaction (CGS) Indicator and Telemedicine Satisfaction Questionnaire (TSQ). The TSQ has previously been shown to have good internal consistency (α = 0.93) for diabetes patients and has since been used to assess patient satisfaction with telemedicine in a genetics setting [18,19]. The CGS has previously shown an excellent internal consistency (α = 0.913) in English when tested at 13 clinical genetic sites at 7 institutions [20]. These were modified to be relevant for our study; items 4, 5, 7 and 10 were removed from the TSQ. Patient satisfaction was indicated using a 1–5 Likert scale response mode, with higher scores indicating greater satisfaction. Participants were also asked to indicate their preference of (1) having their consultation conducted remotely rather than in person at MEH and (2) their preferred method of remote contact either via telephone or video call.

STATA V.15 was used to analyse demographic and survey data. Fischer’s exact test was used to compare independent categorical variables with two categories and Pearson’s Chi squared test was used to compare independent categorical variables with greater than two categories. *P* values less than 0.05 represent results of statistically significant tests.

## 3. Results

A total of 56 carers of children completed the pre-pandemic survey on virtual consultations; 57% were attending new visit appointments and 43% were follow ups (Table 1). Sixty-five percent preferred to have their tests carried out locally. Sixty-four percent of participants (*n* = 34/53) indicated that they would prefer to have a virtual consultation for the review of any results. The majority of families indicated a preference for a telephone call (*n* = 14/31, 45%), followed by written communication (10/31, 32%), with the fewest responses for video call (*n* = 8/31, 26%). In terms of frequency of contact, only 5 participants (9%) opted to be seen virtually for every appointment until a treatment or trial became available.

**Figure 1:**
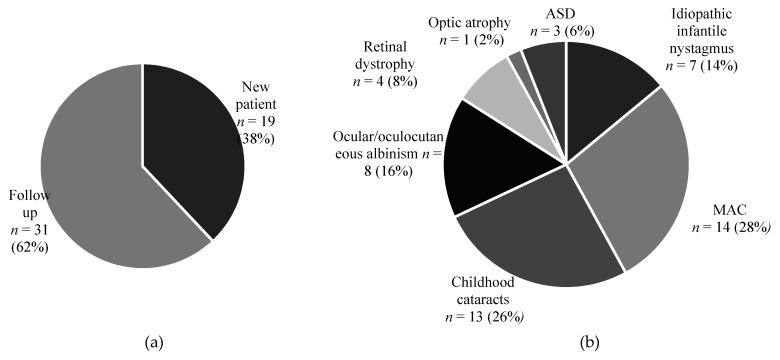
Proportion of patients attending new or follow up appointments and their ocular conditions. (**a**) Thirty-one patients were attending follow up appointments. (**b**) Survey participants were carers of children with the following conditions: Microphthalmia, anophthalmia and coloboma (MAC) were the most prevalent (14 patients), followed by childhood cataracts in 13, ocular and oculocutaneous albinism in 8, idiopathic infantile nystagmus in 7, retinal dystrophy in 4, anterior segment dysgenesis (ASD) in 3 and 1 child had optic atrophy.

During the COVID-19 pandemic remote consultations were implemented for all triaged non-urgent patients. Hence, 50 carers of children (mean age ± SD, 5.5 ± 4.7 years) with a variety of genetic eye diseases (Figure 1) who had received a telephone consultation as part of their standard care completed patient satisfaction questionnaires, with 19 (38%) and 31 (62%) participants attending new and follow up appointments, respectively. Four carers declined to participate, giving a response rate of 93% (50/54).

**Figure 2:**
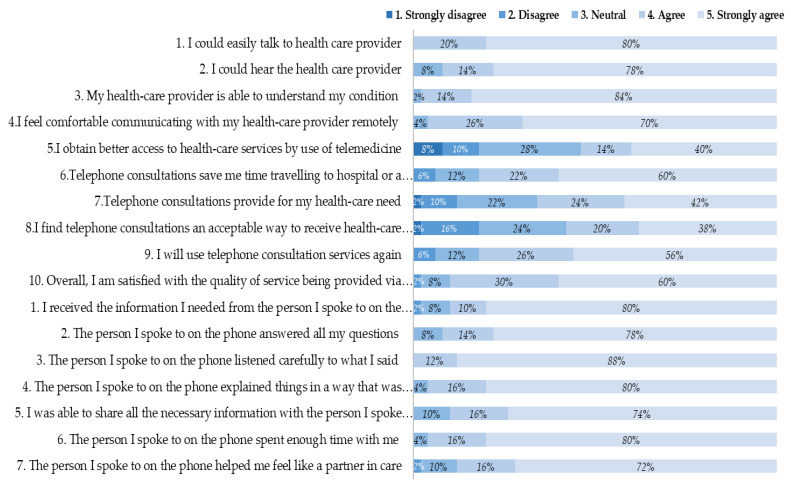
Participants were asked to rate their agreement to statements modified from the Clinical Genetics Satisfaction (CGS) indicator and Telemedicine Satisfaction Questionnaire (TSQ) on a Likert scale (1 = strongly disagree, 2 = disagree, 3 = neutral, 4 = agree, 5 = strongly agree).

Overall, 58% of participants (*n* = 29/50) found telephone consultations to be an acceptable way to receive health-care services, with 24% indicating neutrality and 18% finding it unacceptable (16% disagree, 2% strongly disagree, *n* = 9/50) (Figure 2). However, 67.5% of participants (*n* = 27/40) preferred to be seen face to face rather than remotely. Two thirds of participants (*n* = 33/50) agreed that telephone consultations provided for their healthcare needs with only 12% (*n* = 6/50) indicating that it did not. Ninety-six percent of participants (*n* = 48/50) felt comfortable communicating remotely. All participants agreed that they could easily talk to their health care provider on the phone, 92% (*n* = 46/50) agreed that they could hear them and 98% (*n* = 49/50) agreed that the health care provider could understand their condition. Over half (54%, *n* = 27/50) of participants felt that they obtained better access to care via telemedicine (28% neutral). When asked if telephone consultations saved them time travelling to hospital or a specialist clinic, 94% indicated it would save them time. 

Three participants indicated that they would not use telephone consultations again. All three also found telephone consultations an unacceptable way to receive services and preferred face-to-face consultation; two did not feel that it improved their access to care and one was unsatisfied with the quality of the telephone service. However, none indicated that they felt uncomfortable communicating remotely, and when given the option of telephone or video consultation, all three indicated that they would prefer video consultation over telephone. Overall, 64.3% of participants would have preferred video over telephone consultations.

Patient satisfaction with genetic consultation was generally positive. All participants felt they were listened to carefully. Ninety percent of participants (*n* = 45/50) felt that they received the information they required and were able to share all the necessary information, and 92% (*n* = 46/50) felt that the person they spoke to answered all their questions. Ninety-six percent (*n* = 48/50) felt that the person spent enough time with them and that things were explained to them in a way they could understand. Two negative responses were indicated in total: one person felt that they did not receive all the information they required and one person did not feel like the person they spoke to on the telephone made them feel like a partner in care. 

## 4. Discussion

This is the first patient survey to canvass the opinion of remote consultations prior to implementation and also to evaluate the acceptability of remote telephone consultations to carers of children with genetic eye disease. These conditions are a leading cause of certifiable blindness, accounting for over 10% of sight impaired and severe sight impaired registrations. They often have mobility issues, with 40% not being able to make all the journeys that they want or need to make [21]. The pre-pandemic responses from our survey suggest that the majority of participants are happy to have virtual consultations with their investigations performed locally rather than having to travel to a specialist centre and the preferred virtual mode of review were telephone calls.

Post-implementation, participants found telephone consultations acceptable and they obtained better access to care but many would still prefer to have face-to-face contact with their health care provider. For most genetic eye diseases, establishing the genetic diagnosis can take over a year in a significant number of cases, there are no approved treatments, and patients are kept under long-term follow-up for monitoring disease progression. It is important to emphasise that these patients do require a physical examination, especially all new patients, as 60% of genetic eye disease may be associated with systemic features (which can be overlooked) and this will guide clinical management strategies. But where a clinical diagnosis is established, especially for isolated ocular disorders such as non-syndromic inherited retinal dystrophies, retinal imaging is so advanced, ophthalmologists can utilise this to guide disease progression that may correlate with reported history. With the advent of colour fundus photos and OCT now being available in high-street opticians, shared care may form the future direction for such patients who are stable in long-term follow-up interspersed with virtual and face-to-face specialist consultations. 

Three participants indicated that they would not use telephone consultations again (one new and two follow up patients). Although only a small proportion, this is particularly concerning during the pandemic when access to face-to-face services are reduced. However, it is important to note that these were telephone consultations, and when asked, these same participants preferred video rather than telephone consultations. A survey conducted in the US during the pandemic of 219 adult patients receiving video consultations for routine and acute ophthalmology review (42% response rate) found that nearly half of patients would have delayed seeking care in the absence of a virtual option [22]. Video consultations were also highly rated with 78% stating they would consider participating in a video visit as an alternative to a face-to face encounter in the future. Similarly, a retrospective analysis of telemedicine across 40 specialties in a single New York based centre has also shown high patient satisfaction during the pandemic but found that younger, females and “new visit” patients had lower satisfaction scores [23]. However, we did not find significantly different questionnaire responses between carers attending new versus follow-up appointments. This highlights the importance of evaluating the acceptability of newly implemented services that are intended to increase access to care. Where possible, service users should be included in their development and provided with a choice of contact options. This will avoid missed appointments that could result in suboptimal patient care and waste of health-care resources. Pre-pandemic evaluation of non-genetic adult teleophthalmology services has shown high levels of patient satisfaction [24]. There are no studies relating to ophthalmic genetics, however a study involving 225 participants completing an online questionnaire on acceptability and feasibility of information and communication technologies (ICTs) in the delivery of a cancer genetics service in Wales found them highly acceptable. They did not consider genetic counselling via telemedicine superior to a face-to-face consultation, but they could see how it may benefit those unable to travel [25]. A study conducted at the Mayo Clinic Biobank administered 1200 participants a questionnaire asking how they would like to receive theoretical results using three vignettes (cystic fibrosis, hereditary breast cancer and a pharmogenomics vignette). They found that although 60% of participants reported liking e-visits, the option of receiving results face-to face scored more highly [26].

Evaluation studies of the acceptability of online genetic consultations have been previously conducted, where participants were asked to rate the remote service they received. A study of 54 pre-symptomatic patients in the cardiogenetic and oncogenetic services in the Netherlands receiving online genetic counselling found that patients were significantly more satisfied with their counsellor and counselling session than the control group who received face-to-face counselling in the hospital, but overall only one-third of patients consented to this form of virtual contact [19]. A systematic review of 12 studies in the United States, Canada, the UK and Australia using telemedicine in clinical genetics clinics showed high levels of patient satisfaction and suggested that it has the potential to evaluate paediatric patients with suspected genetic conditions [27].

There were some limitations in this study. This was a relatively small sample size (although it does involve patients with rare inherited eye diseases), drawn from a single centre based in central London. We only included patients who were already at the hospital attending a face-to-face appointment, which may have selected for a cohort with better access to tertiary care. We evaluated telephone appointments only as this was the most popular option indicated on our pre-pandemic survey. Our findings may be reflective of carer perceptions during a prolonged pandemic where anxiety about their child’s condition may be heightened due to cancelled or postponed outpatient appointments. In addition, we found a shift in preference from telephone to video consultations over the course of the study. This finding is likely due to the monumental digital switchover that occurred during the pandemic, increasing user accessibility and familiarity with video communication platforms [28].

## 5. Conclusions

The expansion of ocular genomic medicine and existing pressure on ophthalmology services combined with the current global pandemic means that now more than ever, alternative models of patient care need to be adopted. Measures to enable the continuation of routine and urgent health care delivery during and after the pandemic must be acceptable to patients. Genetic eye disease clinics are suitable for remote delivery and we have demonstrated that they are acceptable to families of children with inherited eye disorders.

## Figures and Tables

**Table 1:** Summary of results on virtual consultation questionnaires from paediatric (carers) respondents. No significant difference (*P* ≤ 0.05) in responses was detected between patients attending initial or review appointments.

	Paediatric Clinic Respondents			
	New	Follow-Up	Total	*P =*
If there was the opportunity to have your tests carried out at a hospital closer to home to avoid travelling to Moorfields and waiting in clinic, would you prefer this?				
Yes	21	14	35	0.77
No	10	9	19	
If there was an opportunity to have future appointments “virtually” – where you would have your tests (scans, blood tests etc.) carried out locally and a Moorfields doctor would review these and contact you with the results, would you prefer this?				
Yes	21	13	34	0.57
No	10	9	19	
If yes, would you prefer to have these review discussions:				
Telephone call	10	4	14	0.51
Video call	6	2	8	
Letter	5	5	10	
If there was the option to be seen “virtually” until a treatment or trial becomes available, would you prefer this?				
Yes	3	2	5	1.00
No	28	21	48	

## Data Availability

Data provided on request.
